# A Novel Missense Mutation in Oncostatin M Receptor Beta Causing Primary Localized Cutaneous Amyloidosis

**DOI:** 10.1155/2014/653724

**Published:** 2014-06-26

**Authors:** Marjan Saeedi, Azadeh Ebrahim-Habibi, Alireza Haghighi, Fariba Zarrabi, Mahsa M. Amoli, Reza M. Robati

**Affiliations:** ^1^Skin Research Center, Shohada-e-Tajrish Hospital, Shahid Beheshti University of Medical Sciences, Tehran 1989934148, Iran; ^2^Biosensor Research Center, Endocrinology and Metabolism Molecular-Cellular Sciences Institute, Tehran University of Medical Sciences, Tehran 1411413137, Iran; ^3^Department of Genetics, Harvard Medical School, Boston, MA 02115, USA; ^4^Department of Medicine and the Howard Hughes Medical Institute, Brigham and Women's Hospital, Boston, MA 02115, USA; ^5^Endocrinology and Metabolism Research Center, Endocrinology and Metabolism Research Institute, Tehran University of Medical Sciences, Tehran 1411413137, Iran

## Abstract

Primary localized cutaneous amyloidosis (PLCA) is a chronic skin disorder, caused by amyloid material deposition in the upper dermis. Autosomal dominant PLCA has been mapped earlier to pathogenic missense mutations in the *OSMR* gene, which encodes the oncostatin M receptor ß subunit (OSMRß). OSMRß is interleukin-6 family cytokine receptors and possesses two ligands, oncostatin M and interleukin-31, which both have biologic roles in inflammation and keratinocyte cell proliferation, differentiation, and apoptosis. Here, we identified a new *OSMR* mutation in a Kurdish family for the first time. Blood samples were taken from all the affected individuals in the family. DNA extraction was performed using salting out technique. Primers were designed for intron flanking individual exons of *OSMR* gene which were subjected to direct sequencing after PCR amplification for each sample. Sequencing showed a C/T substitution at position 613 in the proband. This mutation results in an L613S (leucine 613 to serine) amino acid change. The identified mutation was observed in all affected family members but not in 100 ethnically matched healthy controls. Elucidating the molecular basis of familial PLCA provides new insight into mechanisms of itch in human skin and may lead to new therapeutic targets for pruritus.

## 1. Introduction

Primary localized cutaneous amyloidosis (PLCA MIM 105250) is often seen by dermatologists as a common itchy skin disease. This disorder usually presents with pruritus, skin hyperpigmentation, and thickening (lichenification) but with no systemic involvement. The histological characteristics of PLCA include fibrillary degeneration of basal keratinocytes with increased apoptosis, disruption of dermal unmyelinated nerve fibers, and accumulation of melanosomes in dermal macrophages and Schwann cells. The amyloid in PLCA is derived from keratin after epidermal damage and keratinocyte apoptosis and probably reflects a combination of degenerated keratin filaments and deposition of serum amyloid P component and immunoglobulins [1–3]. PLCA has been reported in association with connective tissue diseases like systemic lupus erythematosus as well as multiple endocrine neoplasia type 2A [1, 4]. Most PLCA cases are sporadic but the disorder is more common in certain parts of the world, including South America and Asia and Middle East, where up to 10% of cases may be familial with an autosomal dominant pattern of inheritance [1]. Familial aggregation and different racial propensity propose that the genetic factors may have a role in the pathogenesis of PLCA.

OSM is a proinflammatory cytokine that is produced by activated monocytes and T lymphocytes and shares significant similarities with members of the IL-6 family of cytokines including IL-6, IL-11, and granulocyte colony-stimulating factor and many of its biological functions are also shared with leukemia inhibitory factor (LIF) [5–7]; these cytokines are multifunctional protein involved in immunity, hemopoiesis, bone modeling, and inflammatory processes. Two types of OSM receptor complexes exist: type I complex is composed of the gp 130 and the LIF receptor ß subunits and type II complex is composed of gp 130 receptor chain and OSMRß chain. Human keratinocytes express a functional type II OSMR on their surface and OSM is able to directly trigger keratinocyte activation and differentiation via the activation of the STAT3 pathway. The OSM-induced signaling cascade also involves activation of certain Janus kinase (JAK1, JAK2, and Tyk2) as well as MAPK pathways [8–10]. OSMRß can also be recruited by IL-31. IL-31 receptor A (IL-31RA) is related to gp130, the common receptor of the IL-6 family cytokines. Recent studies have shown that IL-31RA forms a functional receptor complex for IL-31 together with the beta subunit of oncostatin M receptor (OSMRß). IL-31 might be involved in controlling keratinocyte differentiation and proliferation and also has a number of effects that point to a role in the regulation of immune responses in skin [8, 11]. Pathogenic mutation in oncostatin M receptor (*OSMR*) gene has been identified in PLCA [1–3].

Here, we report the first Kurdish family with PLCA and investigate the clinical features and genetic basis of the disease in this family.

## 2. Materials and Methods

### 2.1. Patients

**Figure 1:**
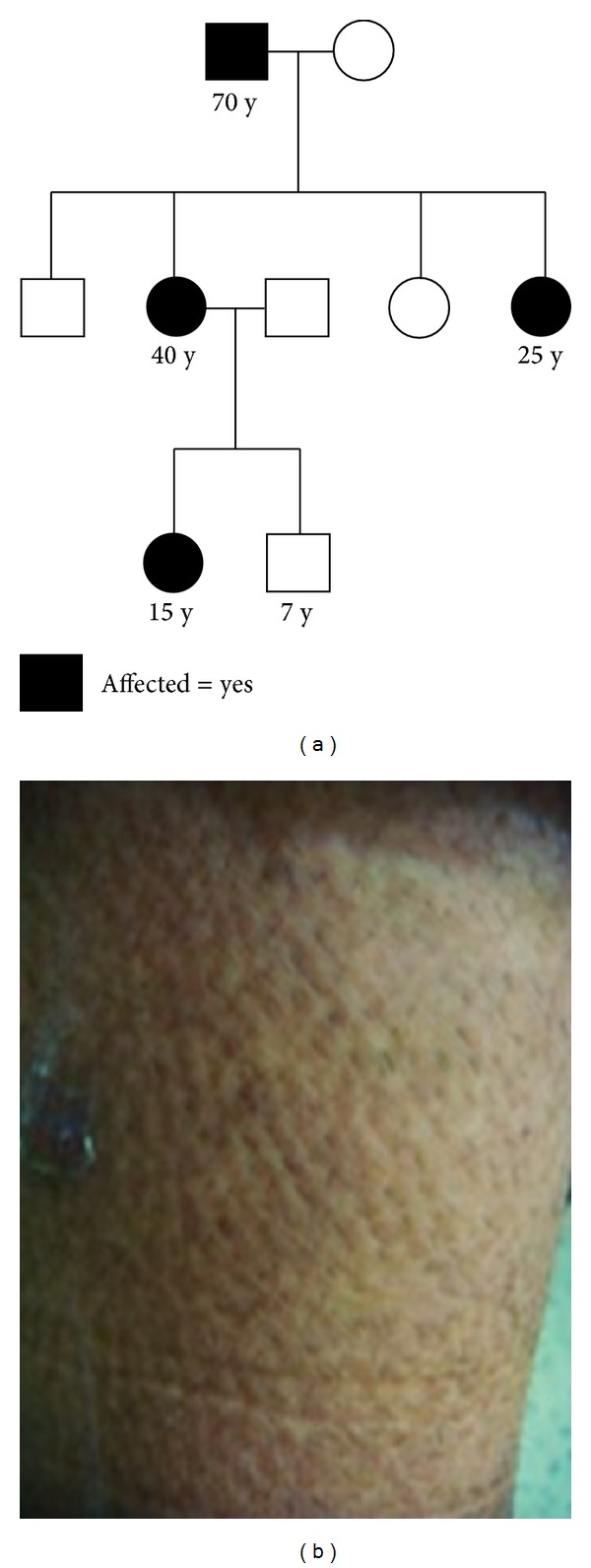
Primary localized cutaneous amyloidosis. (a) Family pedigree; (b) clinical Image of the leg of the affected daughter.

After approval of the study by the Ethical Committee, a written consent was obtained from all subjects, in compliance with the Helsinki declaration. Four biopsy proven PLCA patients from a Kurd family in three consecutive generations (father, two daughters, and one granddaughter) were enrolled in our study; the patients had chronic pruritus and skin hyperpigmentation without any systemic involvement. The disease was more severe in the granddaughter and started earlier (Figure 1). Genomic DNA was extracted from peripheral blood samples using salting out technique [12]. Primers were designed for intron flanking individual exons of* OSMR* gene as described previously [1] which were subjected to direct sequencing after PCR amplification for each samples.

### 2.2. Mutation Screening in Normal Healthy Controls Subjects

In order to rule out the presence of observed mutation in normal population, an assay was utilized for large scale mutation detection using PCR-RFLP technique. After PCR amplification of mutation flanking region (primers sequences are available upon request), the PCR product length generated was 154 bp which after digestion using Bcl*I* restriction enzyme yielded 154 bps of uncut fragment for the TT genotype and two fragments of 154 and 132 bps for the CT genotype.

Mutation screening was performed on 100 normal individuals.

### 2.3. Protein Modeling

The amino acid sequence of the predicted fibronectin type III domain (FNIII domain) of human OSMR, spanning from residue 430–740, was submitted to the PSIPRED server (http://bioinf.cs.ucl.ac.uk/psipred/), and a three-dimensional model of the protein was obtained from the Bioserf module of this server [13].* In silico* mutation induction, further minimization of the native and mutated structures, and interactions visualization were done by the use of MOE 2012.10 (Molecular Operating Environment (MOE), 2012.10; Chemical Computing Group Inc., 1010 Sherbrooke Street West, Suite no. 910, Montreal, QC, Canada H3A 2R7, 2012). The protein BLAST tool of the NCBI server (http://blast.ncbi.nlm.nih.gov/) was used to compare the human OSMR with other species protein.

## 3. Result

**Figure 2:**
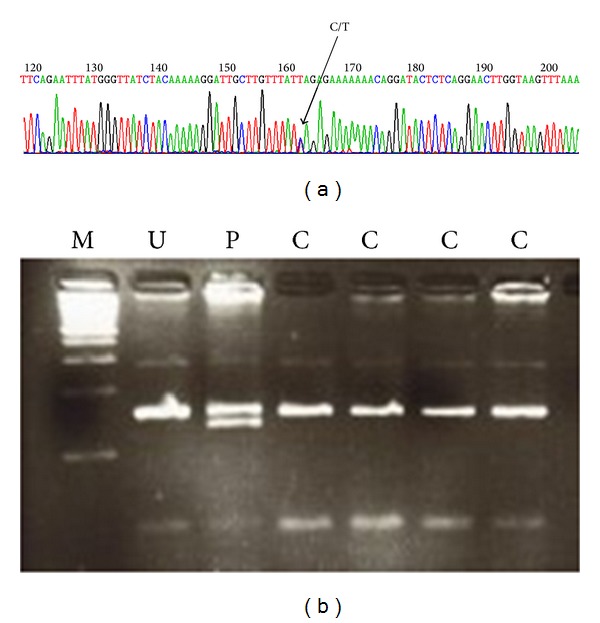
Primary localized cutaneous amyloidosis. (a) The chromatogram shows the single nucleotide mutation in patient with Macular amyloidosis. The C/T substitution in exon 12 of* OSMR* gene causing L613S (leucine 613 to serine) amino acid transition was observed in all affected family members and was absent in normal controls. (b) Gel electophoresis [M = marker U = undigested, test control P = proband, digested C = control, normal individual].

Molecular analysis identified a single nucleotide mutation in the proband, a C/T substitution in exon 12 of* OSMR* gene. This mutation results in a leucine to serine amino acid change at position 613 (L613S). This mutation was present in all affected family members, whereas none of healthy controls carried it (Figure 2).

**Figure 3:**
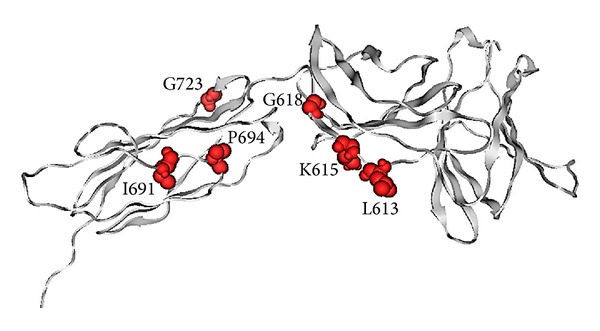
A model of FNIII domains shown with grey cartoons. Reported mutations of OSMR which are related to PLCA are shown in spacefill representation.

**Figure 4:**
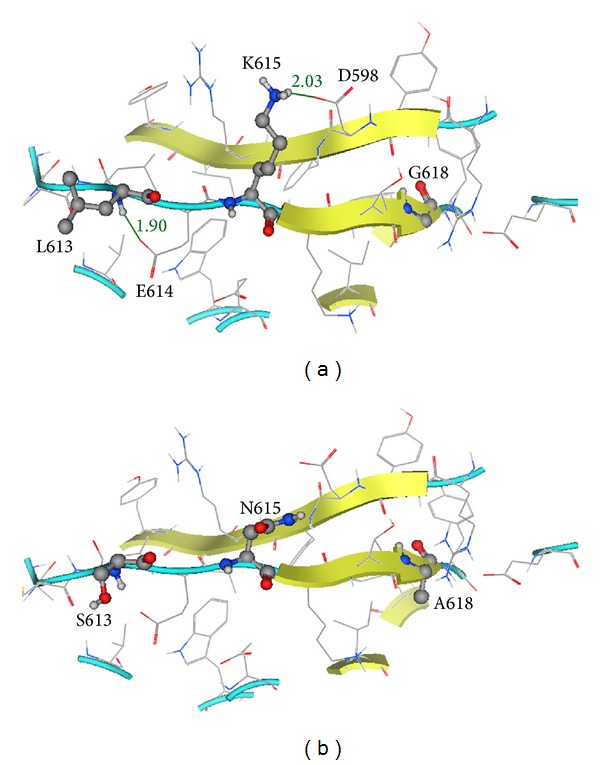
(a) Ball and stick representation of L613, K615, and G618 on the second domain of FNIII. The length of the putative hbonds formed between L613-E614 and K615-D598 are indicated in (Å). (b) Positioning of mutated residues S613, N615, and A618 on the second domain of FNIII.

Previously reported mutations of OSMR that have been related to PLCA include K615N [14], G618A, I691T [1], P694L [15], and G723V [16]. A theoretical model of the three FNIII domains of OSMR was made in order to investigate the possible effect of these mutations. The first two mutations (K615N and G618A) as well as the one that we report here (L613S) are all located on the same strand of the second domain of FNIII (Figure 3). I691, P694, and G723 are positioned in the first FNIII domain (relative to the transmembrane domain and based on schematic representation in Arita et al. study [1]). Residues 613, 615, and 618 are close to each other and their intramolecular interactions may overlap (Figure 4(a)). Two hydrogen bonds (hbond) that are detected for these three residues include a backbone hbond between L613 and the side chain of adjacent E614 and an hbond between K615 and D598 side chains. When observing the residues located in a 4.5 Å space, around these residues, V531, E534, R600, C611, L612, E614, and K615 are found to be potentially interacting with L613, from which R600, E534, and E614 as well as L613 itself are again positioned in the vicinity of K615. Similarly, D598, which has an important interaction with K615, and K616, whose positioning may impact the orientation of K615, are both located in the 4.5 Å area around G618.

A mutation of leucine to serine is an important change from a biochemical point of view; while leucine side chain has mainly the possibility of making van der Waals contacts with its neighbor residues, serine possesses a hydroxyl group with the potential of forming hydrogen bonds with the surrounding solvent or even residues located in the adjacent strand such as R600, thus shifting the original residue pattern of interactions (Figure 4(b)). Furthermore, alignment of the human protein with various species OSMR shows a conservation of this leucine, which is found, for example, in* Pan troglodytes, Odobenus rosmarus divergens, Felis catus, Bos taurus, Equus caballus, Ovis aries, Dasypus novemcinctus, and Pteropus alecto*. K615 and G618 have also been reported to be highly conserved residues [1]. The mutation of lysine (615) to asparagine would directly impact its potential to form an hbond with the D598 of the adjacent strand. Such changes could potentially lead to conformational changes in this domain of FNIII. Finally, the mutation of glycine (618) to alanine would result in the formation of a side chain (although not so voluminous), which may have the potential of making further van der Waals interactions (Figure 4(b)).

**Figure 5:**
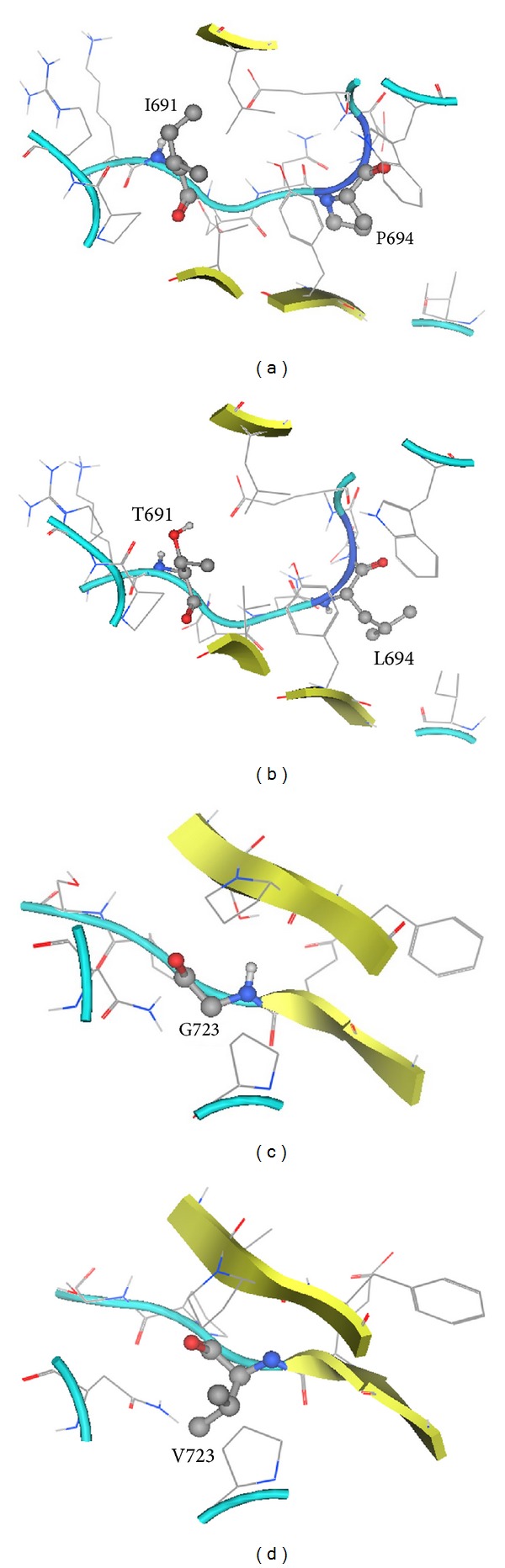
(a) Location of I691 and P694 (ball and stick) on the first domain of FNIII. (b) Positioning of mutated residues T691 and L694. (c) Location of G723 on the first domain of FNIII. (d) Positioning of mutated residue V723.

Of the three residues reported to mutate that are located in the first FNIII domain, I691 and P694 are close to each other and P694 is positioned in a turn (Figure 5(a)). Isoleucine is an aliphatic residue which may be involved in hydrophobic interactions and will be changed to a polar residue upon mutation to threonine. This threonine would then have the potential to make new hbonds, for example, with the adjacent E (696) (Figure 5(b)). Proline is a rigid residue (Figure 5(a)), and a change to leucine would increase the flexibility of the protein structure in this location (possibly affecting the turn conformation) as well as provide a potential to make van der Waals interactions. Glycine is devoid of side chain, and the mutation of G723 (Figure 5(c)) to a valine residue would result in a protrusion from the strand where it is positioned, toward the parallel strand, and there would be a potential for interaction with the neighbor P (625) (Figure 5(d)).

## 4. Discussion

Although the exact pathogenesis of PLCA remains unclear, previous observations suggest a link between the IL-31 and OSM signaling and pathogenesis of PLCA. Overall, the three mutations that occur on residues 613, 615, and 618 of OSMR*β* may all cause some conformational changes in the second domain of FNIII, but their positioning (more or less on the same side of a single strand) is suggestive of their putative direct effect in disrupting intramolecular interactions that are essential in the dimer formation of OSMR. This is in line with the previously proposed theory of Arita et al. in [1] and it may be hypothesized that mutations occurring in other residues located in this strand may also result in deleterious effects. I691T and P694L mutations that are less exposed on the protein surface may affect the conformation of the first FNIII domain, in an intramolecular level, but it should also be mentioned that, based on our model, these are located in a part of the protein which has not a very defined secondary structure composition. The G723V may have similar effects too. In the case of these three mutations, and specially about G723V, based on the positioning of these residues in our model, the effects may be assumed to be exerted by affecting the conformation of the protein itself, and have an indirect effect on the ability of the protein to form heterodimers. This is a theory that has to be confirmed by further experimental evidences.

Mutations involving members of the IL-6 receptor gene family like OSMRß and IL-31RA results in dysfunction of the downstream signals like Jak/STAT, Erk1/2, and PI3K/Akt with antiapoptotic effects in several tumor cell lines and this might also be the reason of keratinocyte apoptosis in PLCA [16, 17]. In addition, skin biopsies of patients with PLCA showed diminished innervations of epidermis and dermoepidermal junction indicating the involvement of neural system in this disease [18]. Both OSMRß and IL-31 RA mRNA are expressed in a subset of small-sized nociceptive neurons of adult dorsal root ganglia and dermis of the skin [19]. OSM plays an essential role in the development of a subtype of nociceptive neurons in the Dorsal root ganglia [18] and IL-31 can stimulate unmyelinated C fibers in the dermis. Decreased function of OSMRß may lead to degeneration of small nerve fibers. Severe pruritus observed in lichen amyloidosis might be the result of the hypersensitivity of the remaining nerve fibres as a response to an unexplained neurodegeneration of the absent nerve fibres [16, 20].

Alzheimer's disease is a neurodegenerative disorder associated with amyloid deposition like PLCA. In these patients, LIF expression was identified in hippocampus and in the temporal cortex, indicating a role for LIF in neuronal damage or repair in these sites [21]. LIF and OSM have significant functional similarities and share type I complex receptor. We can hypothesize that, in PLCA, a functional decrease in OSMRß and IL-31 RA signaling pathway may cause an increase in signaling through type I OSM complex (LIF receptor and gp130), which may lead to a peripheral neurodegenerative condition like Alzheimer's disease. This hypothesis needs to be further investigated.

IL-31 has been shown to be one of the many mediators inducing inflammation and pruritus in atopic dermatitis [22] and its serum level could be used as an objective reliable marker of atopic dermatitis severity in children [23]. L613S in OSMRß causes reduced IL-31 signaling in PLCA with severe pruritus which is contrary to the above findings in atopic dermatitis. This supports that neural components might have a prominent role in the pathogenesis of PLCA [16].

It has been shown that IL-31 can induce the secretion of monocyte chemotactic protein-1 (MCP-1). MCP-1 can attract monocytes, which would differentiate to macrophages at the sites of injury or inflammation. It can be speculated that the amyloid clearance of keratinocytes is compromised in PLCA because of limited recruitment of immune cells to the lesional sites. Deficiency of this scavenger function has been also observed in Alzheimer's disease, which is also characterized by amyloid deposition. Failure of cellular debris clearance from the lesional tissues by the innate immunity may be a common event in these disorders [24]. Recently, diminished level of MCP-1 was identified in PLCA skin [24].

Although PLCA is not life-threatening, the disease affects the patients' quality of life significantly from severe itch to undesirable appearance; therefore, new and efficient therapeutic approaches are important. Additional studies are required to investigate our current hypothesis that can lead to better treatment and management strategies.
